# Elastic waves in particulate glass-rubber mixtures

**DOI:** 10.1098/rspa.2020.0834

**Published:** 2021-05

**Authors:** Kianoosh Taghizadeh, Holger Steeb, Stefan Luding, Vanessa Magnanimo

**Affiliations:** ^1^ Multi-Scale Mechanics, Faculty of Engineering Technology, MESA+, University of Twente, Enschede, The Netherlands; ^2^ Institute of Applied Mechanics (CE), SC SimTech, University of Stuttgart, Stuttgart, Germany

**Keywords:** wave propagation, granular mixture, elastic stiffness, attenuation

## Abstract

We investigate the propagation of waves in dense static granular packings made of soft and stiff particles subjected to hydrostatic stress. Physical experiments in a triaxial cell equipped with broadband piezoelectric wave transducers have been performed at ultrasound frequencies. The time of flight is measured in order to study the combined effect of applied stress and rubber content on the elastic properties of the mixtures. The bulk stiffness deduced from the wave speed is nonlinear and non-monotonic with the increasing percentage of rubber with a more prominent effect at higher pressures. Moreover, in the frequency domain, a spectral analysis gives insights on the transition from a glass- to a rubber-dominated regime and the influence of rubber particles on the energy dissipation. Mixtures with rubber content below 30% show enhanced damping properties, associated with slightly higher stiffness and lighter weight.

## 1. Introduction

The behaviour of particulate mixtures is of interest for a large number of materials, and applications, including sintering, ceramics, gels, mineral processing, pharmaceutics, environmental and geotechnical enginee- ring. In addition, the importance of wave propagation into a granular media comes up in application such as oil exploration, earthquake and roads construction.

In geotechnical engineering, it is common to incorporate recycled materials (e.g. shredded or granulated rubber, crushed glass) into earth constructions for conventional designs and soil improvement projects [1–3]. Similarly, mixtures of asphalt and concrete are widely used to construct roads [4,5].

Exploring the effect of granular composition on the effective physical properties of mixtures can help optimizing industrial processes, and engineering structures [4,6–8]. The topic has received increasing attention in recent years [9–14]. Particular interest has been devoted to the response of mixtures to propagation of elastic waves, and the effect of soft components to dampen high amplitude waves. Despite the massive amount of work on granular mixtures, a deep understanding from a micromechanical perspective is still lacking. In fact, the role of the different components in the mixture can hardly be discerned by classical experiments.

In early experimental works [9–13], investigators have shown that the velocity of shear waves in binary sand-rubber mixtures scales in a nonlinear and non-monotonic fashion with an increasing volume fraction of rubber chips. A similar behaviour was observed more recently in [15] for longitudinal waves in binary mixtures of glass and rubber beads of equal diameter.

Although several methods are commercially available to determine the stiffness of geomaterials, both in the laboratory and in the field, (ultrasound) wave propagation techniques are widely accepted for their rapid, non-destructive, and low-cost evaluation methods. The use of piezoelectric transducers to estimate small-strain stiffness of soils from wave velocity has been well established nowadays [16–22].

Mechanical waves are perturbations moving through space and time in a medium, where the small deformations lead to elastic restoring forces. This causes a transfer of momentum and energy through particle contacts, with little mass transport. The propagation of the mechanical wave through the medium provides valuable effective information about the medium itself. By applying this method to samples, one can deduce their mechanical bulk response [23,24]. For a complete characterization, the dispersive behaviour of the material must be provided, which relates phase velocity with frequency [25,26].

Along with the characterization of the material bulk stiffness and dispersion, wave analysis provides information about the attenuation properties of the medium. When a mechanical wave propagates through a medium, a gradual decay of wave amplitude can be observed. In certain materials, wave amplitude is only reduced by the spreading of the wave, so-called scattering. ‘Scattering’ is the reflection of the sound waves in directions other than propagation due to energy distribution on an expanding wavefront. Another reason for amplitude decay is the energy absorption due to viscous momentum interaction, as related to material properties. The combined effect of scattering and absorption is called attenuation. Intrinsic attenuation caused by viscous case can be experimentally obtained via the spectral ratio method.

In physics, the energy loss (intrinsic attenuation) is usually characterized by the quality factor *Q* defined as the ratio between the energy stored and energy loss per frequency cycle due to viscous momentum interaction. It has long been believed that attenuation is an important quantity for the characterization of particulate systems like sands, rocks and pore fluid properties, e.g. saturation, porosity, permeability and viscosity because the attenuation is more sensitive than the velocity [7,27–30]. Also, the quality factor is a prominent parameter having an important effect upon the amplitude and duration of ground motions during earthquakes [7,30–33]. Its determination appears to be a crucial point for the quantitative interpretation of the amplification effects often produced by surficial deposits [34].

In this study, we apply ultrasound through transmission techniques in order to study stiffness and attenuation in granular materials made of mixtures of glass and rubber beads. The focus is on the effect of the volumetrical composition of the mixture and the stress level, where these factors can be exploited to optimize the material stiffness and density [15]. In addition, granular media show intrinsically high attenuation. Thus, understanding the frequency-dependent attenuation of such materials could potentially be used for inverse material design. The main goal of this article is to enhance the dissipative, elastic and lightweight properties of materials (like soils, asphalt, etc.) by deliberately adding dissipative, soft, and light rubber particles. In other words, to design a granular material showing maximum attenuation *Q*^−1^ in a certain frequency range, along with high stiffness and low density. This allows for a novel design methodology for calm, smooth, and smart materials that can be better in various aspects than their separate components.

This paper is organized as follows: In §2, we describe the details of the experimental set-up; we show the experimentally obtained elastic stiffness of the mixtures in §3; frequency analysis of different glass-rubber mixtures are shown in §4; in §5, we compute the quality factor of particular samples using the spectral ratio method. Finally, §6 concludes the paper.

## 2. Experiments

### (a) Test procedure

The experimental set-up and the applied methodology are described in this section. Glass and rubber particles with similar size (*d*_*r*_ = *d*_*g*_ = 4 mm) are used to prepare cylindrical specimens with different volume fractions of glass and rubber beads. Using similar size particles will isolate results due to the difference in particle properties with regards to their elastic or viscoelastic behaviour. The material characteristics for both glass and rubber beads are reported in table 1; for further information regarding the particles stiffness see appendix A.

**Table 1.** Properties of glass and rubber particles.

used material properties	glass	rubber
diameter (mm)	4	4
mass density (kg m^−3^)	1540	860
Young’s modulus (GPa)	65	0.185
Poisson’s ratio (–)	0.2	0.5

Let *ν* = d*v*_*r*_/d*v* be the volume fraction of the rubber particles (fraction of space possessed by the rubber particles), with the total volume of rubber particles given by d*v*_*r*_ and the total volume of the mixture, d*v*_*r*_ + d*v*_*g*_, given by d*v*. Glass-rubber samples were prepared with variable rubber content, *ν* = 0, 0.1, 0.2, …, 0.9, 1.0, where a mixture with *ν* = 0 is composed of glass particles only and *ν* = 1.0 of rubber particles only. In general, differences in size, density, stiffness and shape of particles could lead to segregation in granular mixtures. Thus, care is taken to prepare homogeneous mixtures and avoid segregation by minimizing vibration during specimen preparation. Each sample was prepared by first mixing particles to ensure that the glass and the rubber beads were evenly mixed. After that, specimens were prepared by allowing particles to fall freely from a funnel into a flexible latex membrane (with 0.304 mm thickness and fit tightly around prepared specimens) stretched by a mold for isotropic tests. Densification is attained by tapping. All granular specimens are tested without any pore liquid, i.e. they are dry. In order to quantify intrinsic attenuation of the sample by the spectral ratio method, two sample heights (100 and 70 mm) are considered in this study.

**Figure 1.**
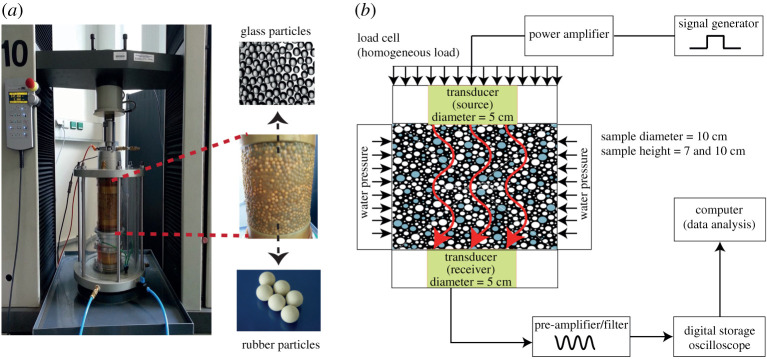
(*a*) Experimental set-up of the acoustic triaxial cell with embedded piezoelectric transducers. A prepared sample is shown with glass (dark) and rubber (light) particles. (*b*) Schematic sketch of the experimental set-up. (Online version in colour.)

The ultrasound instrumentation of the system consists of a pair of 100 kHz *P*-wave broadband piezoelectric transducers (Olympus-Panametrics Videoscan V1011), an arbritrary waveform generator (Tektronix AFG 3101), a broadband power amplifier (E&I 1040L), a pre-amplifier/low-pass filter (Olympus-Panametrics 5077PR) and a digital oscilloscope (PicoScope 5444B). Figure 1*b* shows a schematic drawing of the set-up and peripheral electronics.

A confining pressure *p*^*c*^ and a mechanically applied axial load (Schenck RM 100 with a digital DOLI EDC580 controller) is applied to the sample having a diameter of 100 mm (figure 1*a*). In subsequent stress increments, the granular samples are compressed in axial direction via the piston of the mechanical testing device. We are aiming for hydrostatic stress states. By hydrostatic stress state, we refer to a configuration in which normal stresses are equal and shear stresses are zero, i.e. the loading (axial) and radial directions are principal directions of stress. Such a stress state is also referred to as uniform or isotropic. Thus, at each load step, the confining pressure *p*^*c*^ is adopted to the axial stress state. Since the samples are tested in isotropic state, we express the hydrostatic stress state with the pressure *p*, i.e. *p*^*c*^ ≡ *p*, in this work. Water is used as a confining fluid for the samples enclosed by a rubber membrane. In total, 10 hydrostatic stress states are analysed, between *p* = 50 and 500 kPa. At each stress level, a high voltage burst signal is excited to measure the time of flight. The top plate and the bottom plate of the cell are instrumented with the piezoelectric transducers. The piezoelectric transducers have a diameter of 38 mm (around one-third of the sample diameter) and are in direct contact with a 10-mm thick polymethylmethacrylate contact plate (delay block) with a diameter of 100 mm adjusted to the sample size. We use two identical vertically aligned piezoelectric transducers. The top transducer is acting as ultrasound source while the transducers at the bottom of the triaxial cell acts as receiver. The acoustic sound pressure is small enough to not destruct the granular samples, i.e. rearrangements of the micro-structure (fabric) are prevented. In this configuration, all through-transmission ultrasound investigations have been performed. The transmitted signal is a ±400 V square wave pulse. The signals transmitted and received are pre-amplified, filtered and recorded with a digital oscilloscope (LeCroy WaveSurfer 1 GHz). The signal-to-noise ratio is improved by repetitive averaging of 100 detected signals using the digital oscilloscope and then sent to a computer for further processing. Thanks to a new generation of oscilloscopes, averaging of signals, which are sent into the samples repeatedly, is done automatically. Therefore, a clean (not noisy) signal is generated as an output. Sending electronic signals and averaging them is quite fast (less than 10^−6^ s), which means there is no time dependence in the collected signals.

All experiments performed for a certain rubber content and a stress state are repeated five times in order to avoid configuration-dependent results. We would like to emphasize that repeating the experiment means setting up a new granular packing in the triaxial cell. For each experiment, particles are mixed and poured into the cell again. Later on, when the results are shown, an error bar indicates the standard deviation of the five experimental results.

### (b) Mixture properties

For each rubber content *ν*, the mass density of the sample is given by

2.1 $$\rho_{B}=(1-\nu )\rho_{g}+\nu \rho_{r},$$

where *ρ*_*g*_ and *ρ*_*r*_ are the bulk densities of glass and rubber beads that are calculated at fraction *ν* = 0 and *ν* = 1, respectively. The numbers of particles used in each experiment, *N*_*g*_ (number of glass particles) and *N*_*r*_ (number of rubber particles), are estimated by knowing the weight of particles poured into the chamber and the weight of each particle.

Knowing the cylindrical geometry (volume) of granular sample *V*_box_ = (*π*/4)*D*^2^*H*, with *D* = 100 mm (diameter) of the sample and *H* = 100 mm (height) of the sample, before and after deformation, and volume of every single particle, *V*_bead_ = (2*π*/3)*d*^3^, one can determine the porosity *ϕ* of the sample

2.2 $$\phi =1-\frac{(N_{g}+N_{r})V_{\text{bead}}}{V_{\text{box}}}.$$

**Figure 2.**
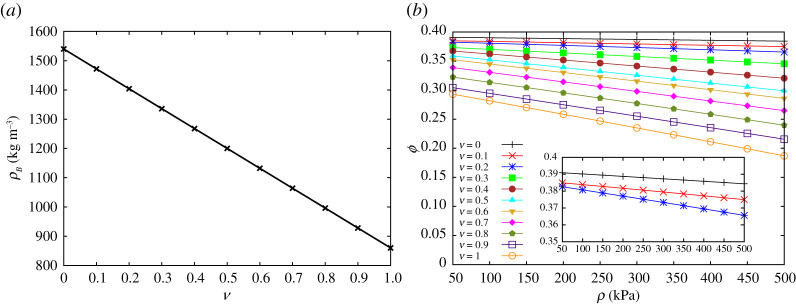
(*a*) Mass density of unconfined samples at different rubber content. (*b*) Porosity versus pressure for samples with different rubber contents. (Online version in colour.)

In figure 2, we plot the mass density at unconfined pressure state versus rubber content and the porosity against applied pressure. Rubber is softer than glass and can deform easily, filling the pores. Therefore, the porosity decreases by increasing both pressure and rubber content. Adding rubber particles makes the system softer. Thus, the porosity becomes sensitive to the change of pressure with the slope of the lines getting steeper for high rubber contents.

## 3. *P*-wave velocity

**Figure 3.**
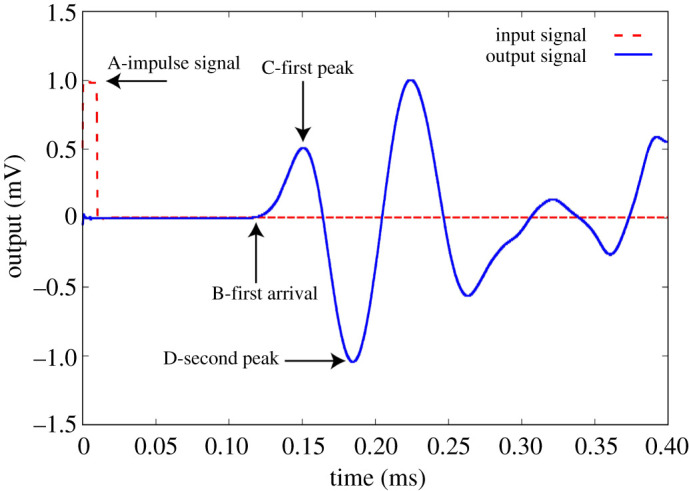
Typical input and output signals from the transmitting and receiving piezoelectric transducers. (Online version in colour.)

In this section, the time of flight of the wave through the samples, for each rubber content and hydrostatic stress state, is measured, which eventually is converted to the bulk stiffness. A typical output is presented in figure 3 for a sample without rubber (*ν* = 0) at pressure *p* = 300 kPa. In highly attenuated granular media, there is always a significant uncertainty and difficulty associated with the determination of the wave travel time (see appendix B). Suggested criteria and recommendations vary depending on the installation, application and input signal. Here, we adopt a consistent peak-to-peak approach [35].

**Figure 4.**
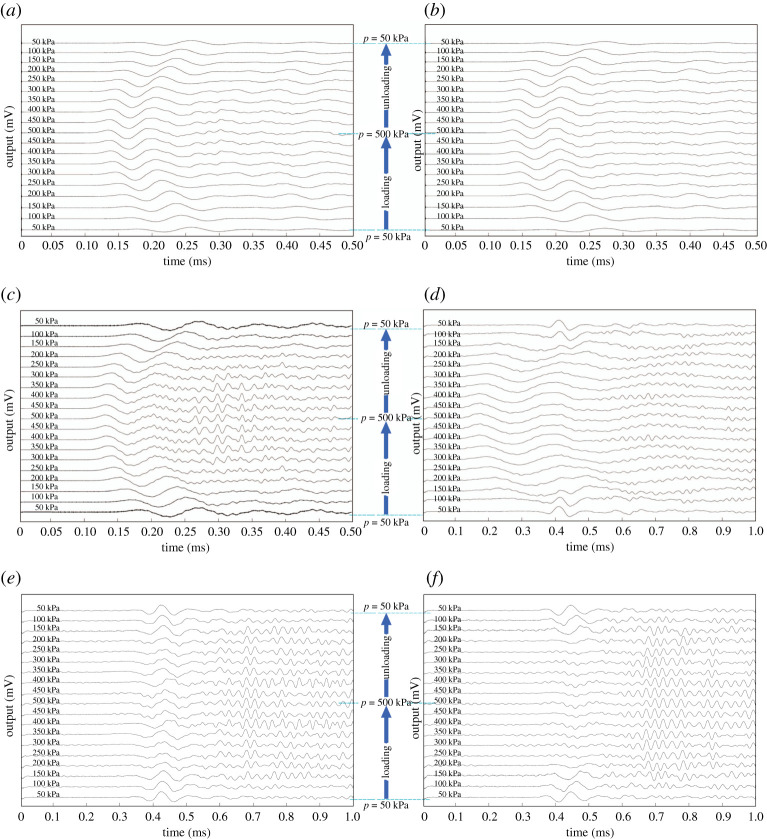
Received *P*-wave signal series during loading (from 50 to 500 kPa) and unloading (from 500 to 50 kPa) for samples with different rubber content, (*a*) *ν* = 0, (*b*) *ν* = 0.1, (*c*) *ν* = 0.3, (*d*) *ν* = 0.5, (*e*) *ν* = 0.7 and (*f* ) *ν* = 1. Note that the limit of the *x*-axis varies for different plots for the sake of visibility. It is considered to be 0.5 and 1 ms for *ν* < 0.5 and *ν* ≥ 0.5, respectively. Note, waves have been normalized by their maximum value. (Online version in colour.)

Figure 4 shows the *P*-wave signals recorded during hydrostatic loading and unloading for several rubber volume fractions. Regarding the acquired signals, it is obvious that the waveforms are sensitive to changes in material composition and applied hydrostatic stress state. For samples prepared with a low rubber content (*ν* = 0, 0.1, to 0.3), the waveforms show a clear peak followed by a deep valley, which moves to the left on the time axis with increasing hydrostatic stress. For *ν* = 0.3, additional features start to appear associated with high-frequency transmission, especially for high-pressure levels. At *ν* = 0.5, the behaviour resembles that of lower rubber contents only at high pressure, while at low pressure a new peak appears later in time, and high-frequency ripples are visible in both the peak and post-peak parts. For *ν* ≥ 0.7, the pressure-dependent peak disappears and high-frequency features dominate. Comparison between *ν* = 0.3, 0.5 and 0.7 clearly shows a pronounced change in the waveforms associated with a transition from stiff to soft dominated regimes. Adding soft particles to the sample not only leads to a delay in the events but also to additional features in the post-peak part of the signal. Furthermore, waveforms of *ν* = 0.7 and 1 reveal that soft dominated samples are insensitive to pressure.

By measuring the travel time of the *P*-wave (*t*_*p*_) and the tip-to-tip distance between transmitting and receiving transducers (*L*), the *P*-wave velocity in the specimen (*v*) is obtained as [36]

3.1 $$v=\frac{L}{t_{p}}.$$

**Figure 5.**
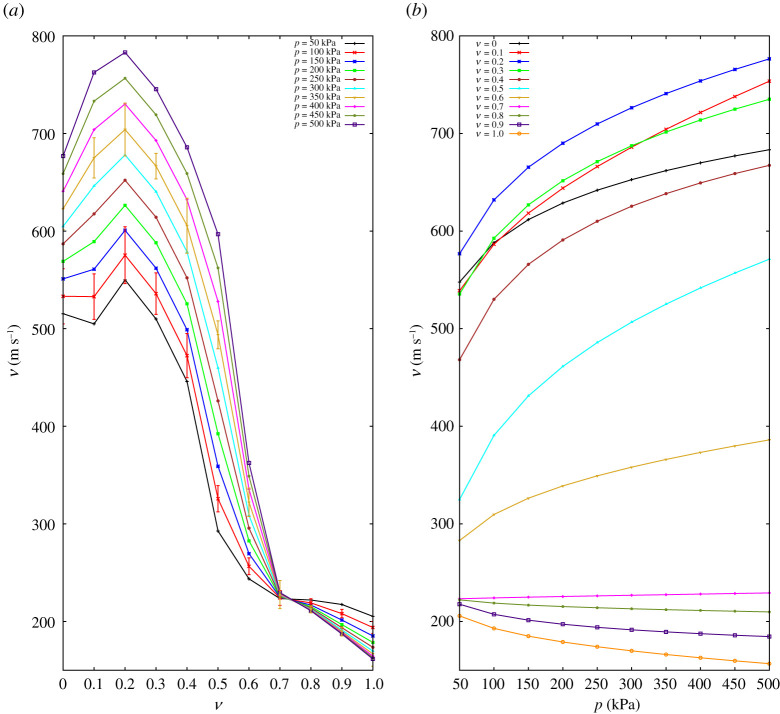
(*a*) *P*-wave velocity against fraction of rubber. Bars on the curves of *p* = 100 and 350 kPa represent the standard deviation of tests repeated five times. (*b*) *P*-wave velocity against confining stress. (Online version in colour.)

Figure 5*a*,*b* shows the wave velocity versus rubber content and pressure. The wave velocity remains fairly constant up to *ν* = 0.3. By increasing the volume of rubber, there is a considerable drop in the wave velocity for all pressure levels, which can be associated with the end of the glass-dominated regime. The wave velocity is again relatively stable for *ν* = 0.7 to 1, where the medium is, eventually, controlled by rubber particles.

In the long-wavelength limit, the longitudinal *P*-wave modulus, *M*, is related to the velocity, *v*, by

3.2 $$M=\rho_{B}v^{2},$$

where *ρ*_*B*_ is the mixture’s bulk density of the sample as given by equation (2.1).

**Figure 6.**
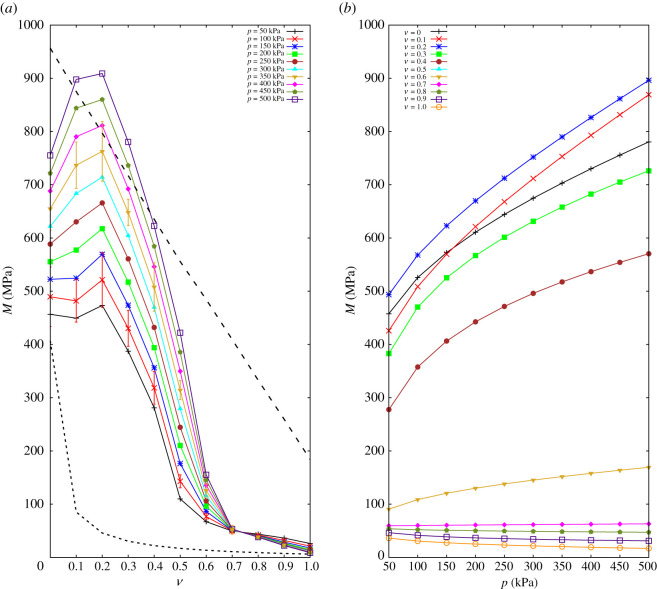
(*a*) Averaged *P*-wave modulus against rubber fraction *ν*. Bars on the curves of *p* = 100 and *p* = 350 kPa represent the standard deviation of experiments repeated five times; the averaged quantities are obtained by averaging the experimental results. Dashed lines in this plot represent lower- and upper-bands of modulus obtained from the theory. (*b*) *P*-wave modulus against hydrostatic stress state. (Online version in colour.)

Figure 6*a* shows the evolution of the *P*-wave modulus with rubber content at different hydrostatic stress states *p*. The *P*-wave modulus, *M*, shows high values for a rubber content up to *ν* = 0.3. In the case of a high hydrostatic stress, adding a small amount of soft particles surprisingly enhances the effective stiffness of the medium and the highest modulus is observed at *ν* ≈ 0.2.

Several arguments may contribute to this interesting observation associated with the high deformability of rubber particles under hydrostatic stress. First, the deformed rubber induces an increase in the number of contacts among glass particles, thus increase stiffness, if an effective medium argument is applied [16,36–38]. Moreover, an increase in contacts stretches and stabilizes the tortuous wave path, resulting in an effectively shorter travelling distance and higher participation of glass-glass contacts in wave transmission. These phenomena are more pronounced at higher hydrostatic stress states. However, it must be noted that the improvement only applies when samples are in the stiff-dominated regime, i.e. *ν* < 0.3.

For 0.3 ≤ *ν* ≤ 0.6, there is a considerable drop in the *P*-wave velocity, which highlights the transition from glass- to rubber-controlled media. The modulus is again relatively stable for 0.6 ≤ *ν*, which is linked to the dominance of the rubber media. In order to compare the measured modulus with the available mixture theory (Hashin-Shtrikman [39–42]), upper and lower bands are added as dashed lines in figure 6*a* for samples with the highest and lowest pressure. Fully glass and rubber samples, *ν* = 0 and 1, show effective moduli in between the two bands predicted by the theory, as expected. However, when mixed samples are taken into account, one can find that the theory fails to estimate the upper limit of samples with rubber content between 0.1 and 0.3.

In figure 6*b*, the *P*-wave modulus *M* is plotted against the hydrostatic stress state expressed in *p*. While the sample with *ν* = 0 shows a different behaviour, the relative increase of the modulus with pressure is similar for mixtures with *ν* ≤ 0.4. The behaviour dramatically changes when rubber content moves from *ν* = 0.4 to 0.5 and 0.6. Further on, at higher rubber contents, the modulus becomes almost independent of pressure. We associated the change in the material behaviour with the transition from a glass-dominated regime, where waves travel through glass-glass contacts to a rubber-dominated case [43], where the packing seemingly behaves as a homogeneous rubber block [44,45].

## 4. Frequency spectra

Looking at a mechanical wave in time-series profile, we can see that the wave signals can be decomposed into two parts, the initial coherent wavefront followed by an incoherent multiply scattered signal also known as ‘coda’. The initial wavefront is of low frequency in nature, opposite to the coda, which contains high frequencies [46]. The coda contains waves reflected multiple times, e.g. from smaller particles, clusters or inclusions [47]. Hence, it provides information at the smaller structures in comparison with the medium. As we are interested in the response of the bulk structure, the coda wave was discarded from the signal processing and analysis. Thus, the initial wavefront used in §3 is also used here to determine the bulk sound wave velocities. Reference is to the so-called first event transmitted before the second peak (i.e. point (D) in figure 3).

In order to understand the transition in the material behaviour as observed in figures 4–6, we study the previous samples in the frequency domain by applying a spectral analysis using a fast Fourier transform (FFT) to the time-domain signals obtained from the experiments [48]. We focus on the first wavefront that determines the *P*-wave velocity (and the effective stiffness of the sample) and discard the contribution of the incoherent coda wave. It is worth mentioning that a filtering function has not been applied so as to not lose any information.

**Figure 7.**
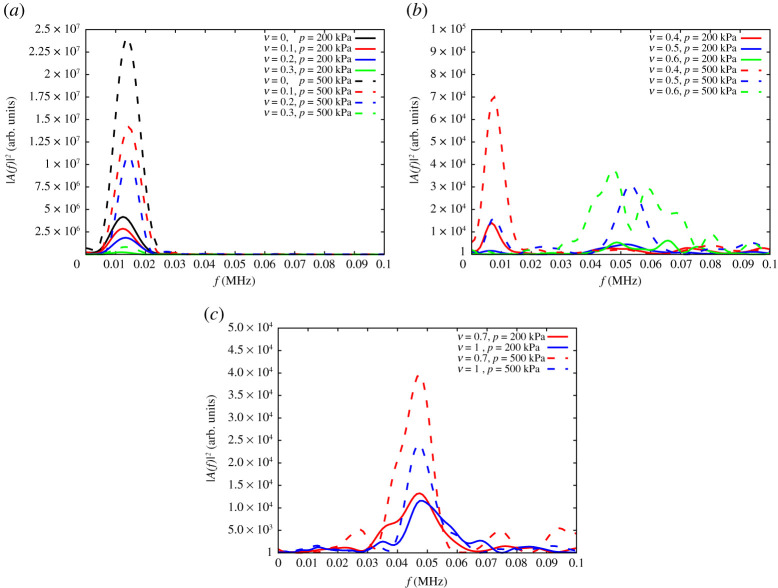
Energy against frequency for samples made with (*a*) *ν* = 0 (black), 0.1 (red), 0.2 (blue), 0.3 (green) rubber content,(*b*) *ν* = 0.4 (red), 0.5 (blue), and 0.6 (green), (*c*) *ν* = 0.7 (red) and 1 (blue), at a hydrostatic stress state of *p* = 200 kPa (solid line) and *p* = 500 kPa (dashed line). (Online version in colour.)

From the spectral analysis of the time-domain signal, we derive the amplitude *A* for a given frequency *f* and thus the Fourier power spectrum |*A*(*f*)|^2^. In figure 7*a*, the spectrum is plotted for samples with rubber contents of *ν* = 0, 0.1, 0.2 and 0.3 at two different hydrostatic stress states of *p* = 200 and *p* = 500 kPa. For these low rubber volume fractions, the main frequency remains unchanged despite the increase in the effective stiffness (figure 6). The dominant frequency does not change with the applied hydrostatic stress, only the power increases with higher hydrostatic stresses.

Furthermore, we focus on the frequency response in the transition regime. Figure 7*b* shows the spectrum against frequency for samples with rubber volume fractions of *ν* = 0.4, 0.5 and 0.6 and two hydrostatic stress levels of *p* = 200 and 500 kPa. For *ν* = 0.4, the main frequency is close to the value obtained for the cases *ν* ≤ 0.3. However, a second peak starts to appear at higher frequency. Samples with *ν* = 0.5 show two peak frequencies, one close to the value seen before, related to glass-driven propagation, and another, very wide at *f* = 0.055 MHz. While the energy associated with *ν* = 0.5 at *p* = 200 kPa is low and widely spread, it becomes clear that at the higher stress state of *p* = 500 kPa the high-frequency signal dominates, but still a low-amplitude, low-frequency peak is surviving. The trend becomes even more pronounced for *ν* = 0.6, where the sample at low hydrostatic stresses shows almost no peak (with the energy possibly stored in the coda) and a bimodal behaviour is appearing at the higher stress state at a frequency about *f* = 0.055 MHz. For these intermediate rubber volume fractions, the first arrival of the *P*-wave (figure 4*d*) seems to be related with the glass network, while the energy is mainly concentrated at higher (rubber-related) frequencies.

The peak observed at *f* = 0.055 MHz is associated with a transitional stage of the system from stiff- to soft-dominated regime. For intermediate rubber contents, *ν* = 0.4, 0.5 and 0.6, the system is controlled by disorganized stiff and soft clusters. The peak at *f* = 0.055 MHz is possibly associated with one (or more) of these clusters, while other, smaller/bigger clusters become dominant when the rubber content changes from *ν* = 0.5 to *ν* = 0.6. Similar transient features, at these intermediate rubber contents, are also related to changes in pressure. In fact, the peak appears when moving from *p* = 200 to *p* = 500 kPa, where it is expected new/more clusters will form. For higher rubber content, larger and organized clusters of rubber particles form and finally behave as a single cluster, i.e. the system is dominated by the soft phase. This is associated with a persistent peak at *f* = 0.055 MHz, which becomes more pronounced with rubber content and pressure.

Finally, rubber contents *ν* = 0.7 and 1 are depicted in figure 7*c*. Unlike the earlier plots, this figure does not show any significant frequency at 0.01 MHz that was associated with glass particles, but the energy is concentrated at 0.05 MHz, that is, the peak frequency of the pure rubber media. Therefore, it can be concluded that glass particles, and therefore the discrete granular packing, do not play an important role in samples with high(er) rubber volume fractions.

It is important to point out that the scale of the *y*-axis is different between figure 7*a*–*c*, meaning that the amplitude in the peak changes by three orders of magnitude from *ν* = 0 to *ν* = 1. Looking at these plots, observations related to energy per frequency lead to an interesting conclusion on the nature of the medium in the different stages. The energy associated with low frequencies is related with the ‘discrete nature’ of the glass beads phase. The low-pass filter associated with particle width, *d* = 4 mm, survives the filtering of the granular medium, which instead traps high frequencies.

This has been observed earlier in [49]. Increasing the amount of rubber particles leads to a more solid/bulk response, rather than discrete, or at least it can be said that the discrete nature is diminished. This can be clearly found by looking at graphs of high rubber content samples where low frequencies do not pass through.

It is worth mentioning that applying the FFT analysis on the input signal gives the dominant frequency of pressure wave at around 100 kHz, which is normal for granular media. The amount of energy exerted by transducers at 100 kHz is approximately 6.6 × 10^−29^ J, which is very small and negligible in comparison with the pressure at which experiments were carried out. As expected, the energy is very small in comparison with the output signals, which ensures that samples have not been burst by the input, i.e. no network rearrangement or particle breakage. In this respect, wave propagation is often referred to as a constant-fabric experiment.

## 5. Intrinsic attenuation of the granular mixture

The purpose of this section is to study intrinsic attenuation of the sample, i.e. the viscous loss of energy during wave propagation with a focus on samples with a low rubber volume fraction of *ν* ≤ 0.3. The attenuation of seismic waves is an important property of the Earth, which is of great interest to material scientists, geo-mechanical engineers and physicists. During acoustic wave propagation, some of the elastic energy is lost (e.g. transforms into heat) per cycle in a propagation waveform through the media. Therefore, we introduce a quantity commonly used in materials science, geomechanics and (geo)physics. The ability of a material to attenuate acoustic waves is measured by the dimensionless quantity *Q* called the quality factor (or sometimes loss factor):

5.1 $$Q=\frac{\text{energy of seismic wave}}{\text{energy dissipated per cycle of wave}}=\frac{2\pi |A(f){|}^{2}}{\Delta |A(f){|}^{2}},$$

where |*A*(*f*)|^2^ is the energy of the wave as introduced in §4, and Δ|*A*(*f*)|^2^ is the change in energy in a single cycle.

Several methods have been developed to compute experimentally the quality factor *Q* [30,50,51]. Here, we apply the spectral ratio method based on the assumption that the ratio of the amplitude at two discrete times, *t*_1_ and *t*_2_ (with the travel distance *H*_1_ and *H*_2_, respectively), varies as a function of frequency during the propagation of the acoustic wave [52–54]. Computation of the spectra of the wavelet and evaluation of the logarithmic ratios for two receivers at depth *H*_1_ and *H*_2_ yields to:

5.2 $$\ln\left|\frac{A_{1}(f)}{A_{2}(f)}\right|=-\frac{\pi (t_{2}-t_{1})}{Q}f+c,$$

where *A*_1_(*f*) and *A*_2_(*f*) are the amplitude spectra at different lengths, *f* is the frequency, *t*_1_ and *t*_2_ are the travel times from the source to the receiver at distance *H*_1_ and *H*_2_, and *c* is a constant which contains all frequency-independent terms like transmissivity, geometrical spreading, source and receiver response, etc. A major strength of the spectral ratio method is that any frequency-independent scaling factor, as the geometrical spreading, falls into the intercept term of the linear regression, *c*, thus, it does not affect the quality factor.

Then the quality factor *Q* factor can be estimated by fitting a straight line to the logarithmic spectral ratio over a finite frequency range. *Q* is directly related with the slope, *m*, of the best-fit straight line as

5.3 $$Q=\frac{-\pi (t_{2}-t_{1})}{m}.$$

The above derivation is the basic idea of the classic spectral ratio method, which is originally derived for the application to vertical seismic profile data [55]. It must be mentioned that even when the data are free of noise, the estimated *Q* can significantly deviate from the true value because the selection of the first peak from the received noisy signal is hard.

**Figure 8.**
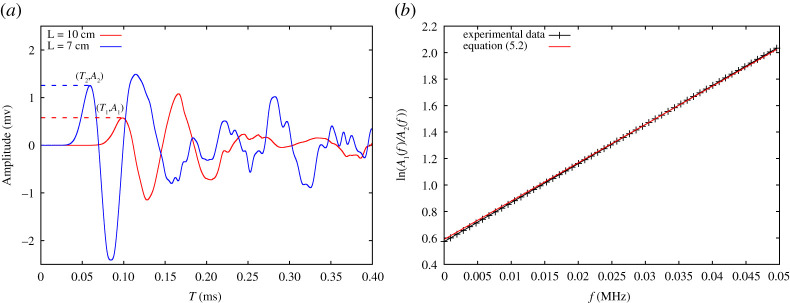
(*a*) Signals recorded for samples with two different lengths (7 and 10 cm) at the same rubber content (*ν* = 0.2) and the same pressure (*P* = 500 kPa). Peak amplitude of the signal for the short sample is *A*_2_ and for the long sample is *A*_1_, and are marked by ‘square’ and ‘circle’, respectively. (*b*) Logarithmic amplitude ratio of two given signals in frequency domain fitted by equation (5.2). (Online version in colour.)

Figure 8*a* shows raw signals recorded in the time domain for two samples with the same rubber fraction at the same hydrostatic stress state but having different sample heights, namely *H*_1_ = 100 and *H*_2_ = 70 mm. It is not surprising to see the shift of the signal to the left side of the time axis for the case of shorter samples, *H*_2_, i.e. signal arrives earlier. From the time domain plot (figure 8*a*), the difference between the time of flight (*δt* = *t*_2_ − *t*_1_) of the two samples is obtained, i.e. the first peak difference, where the first peak is considered since *Q* is measured for a single cycle consistently. To measure *Q*, the signals must be taken from the time domain to the frequency domain by the aid of FFT. After that, the quality factor is measured by fitting a line (equation 5.2) to the logarithmic spectral ratio over a frequency range, *f* < 0.05 [MHz]. From figure 7*a*, it is reasonable to assume that the frequency range to be considered in the measurement is *f* < 0.05 [MHz], as frequencies above this range carry negligible energy (figure 8*b*).

**Figure 9.**
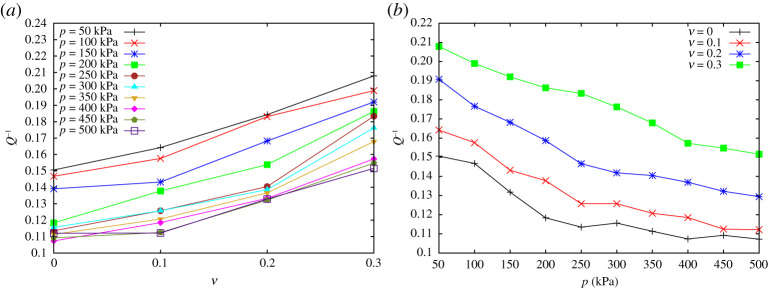
Attenuation factor *Q*^−1^ versus (*a*) rubber contents and (*b*) pressure level. (Online version in colour.)

Intrinsic attenuation is expressed by the inverse quality factor *Q*^−1^ and is plotted against rubber volume fractions (*ν* ≤ 0.3) in figure 9*a* for different pressure levels. When the amount of rubber increases, the quality factor parameter *Q*^−1^ increases in a linear fashion, i.e. the mixture behaves more dissipatively when the amount of soft inclusions is higher. On the contrary, increasing pressure leads to a decrease of damping as shown in figure 9*b*, where *Q*^−1^ is plotted versus the confining pressure.

Figure 9 shows how the quality factor scales in a linear fashion, not only with respect to pressure, but also with the rubber content in the range of *ν* considered here. Combining the results in figures 2, 6 and 9, we summarize that adding roughly 20% of soft inclusions strongly improves the damping of the mixture (about 30%), but also increases its stiffness (up to 15%) and, additionally, provides a much lighter sample (about 15%). Such effective acoustic behaviour of binary mixtures can obviously not be predicted by the application of simple mixture rules.

## 6. Conclusion

In the present experimental investigation, acoustic wave propagation experiments at ultrasound frequencies have been performed to examine the behaviour of biphasic granular mixtures consisting of glass (stiff) and rubber (soft) beads. Acoustic signals inferred from transmission tests using broadband piezoelectric transducers were acquired and interpreted to infer both effective *P*-wave modulus (stiffness) and intrinsic attenuation of the biphasic mixture. The experimental data indicate that the ‘stiff’ skeleton composed out of glass beads controls the effective mechanical response at small rubber fractions, *ν* ≤ 0.3, while the ‘soft’ rubber skeleton prevails at larger rubber volume fractions of *ν* ≥ 0.7. There is a considerable drop in the *P*-wave modulus *M*, only at intermediate mixtures (0.3 < *ν* < 0.6), where the transition from a stiff to soft regime occurs. Interestingly, we found that waves propagate faster in a range of *ν* ≈ 0.2 compared with a mixture composed only of glass beads, *ν* = 0. Such an effective stiff behaviour, which could not be explained by classical mixture rules or effective medium theories, could be observed at all hydrostatic stress levels, but is enhanced by high pressure. The relevant feature can be explained in many industrial applications where processes and bulk material properties have to be optimized.

Furthermore, the frequency spectra, obtained from FFT, show that the dominant frequency for fixed rubber volume fractions is independent of the applied hydrostatic stress, while the majority of the elastic energy moves from low to high frequencies when the rubber fraction is increasing in a small range from *ν* = 0.4 to *ν* = 0.5, with a new sharp peak appearing. Comparing the peak frequencies, the transition from stiff (discrete mixtures) to soft (continuum-like) media was clearly observed.

Finally, intrinsic attenuation in samples composed of low rubber volume fractions was determined. As expected, a systematical increase of viscous energy loss with increasing rubber content was attained, while an increasing hydrostatic stress state reduces attenuation.

The relationship between the *P*-wave modulus *M* and the inverse quality factor 1/*Q* for samples with low rubber volume fractions reveals interesting phenomena: although the stiffness remains almost unchanged with increasing rubber volume (or even increases slightly), the damping increases continuously. Especially, an optimal mixture was attained for a rubber volume fraction close to *ν* = 0.2 at high hydrostatic stress states, showing the highest *P*-wave modulus and significantly high attenuation.

Future work includes the experimental investigations of *S*-waves in glass-rubber mixtures and numerical simulations (using discrete element methods and/or finite-element methods) to reproduce the behaviour of glass-rubber mixtures tested experimentally and gain more micro-mechanical insights.

## Figures and Tables

**Figure 10.**
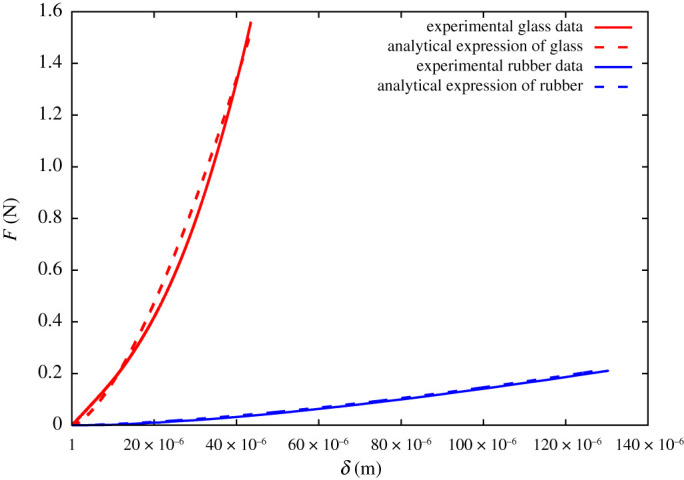
Experimental (solid line) force–displacement plot of a single particle compression fitted by a nonlinear Hertzian law (dashed line). Red and blue colours indicate glass and rubber particles with the properties given in table 1, respectively. (Online version in colour.)

**Figure 11.**
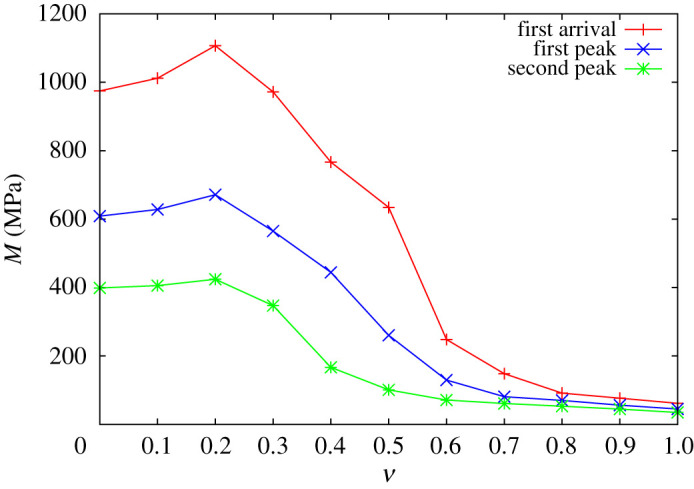
*P*-wave modulus versus fraction of rubber obtained by the first deflection, first peak and second peak points. (Online version in colour.)

**Figure 12.**
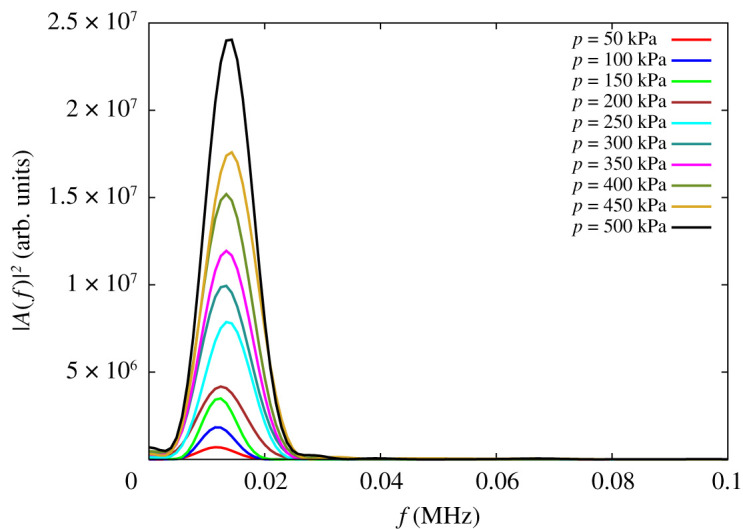
Energy against frequency for a sample made with glass particles, *ν* = 0, at different confining pressure. (Online version in colour.)

## Data accessibility

Experimental raw data, post-processing scripts and post-processed data are freely available under the 4TU General Term of Use License and can be downloaded from the 4TU.researchData repository: https://doi.org/10.4121/14096491.

## Appendix A

To obtain the (elastic) bulk properties of the single glass and rubber particles, we performed a compression test on single particles. The force–displacement curve of glass and rubber is plotted in figure 10. The Hertzian law fits well with the data in the pressure regime considered [56,57]. Starting from the fitting curves, Young’s modulus for glass and rubber particles can be deduced.

## Appendix B

Several difficulties such as the selection of travel distance, the determination of travel time, and near field effects affect the measurement of *P*-wave velocities. These issues have been addressed in [35,49,58–60]. Once these boundary and scale effects are evaluated and their effects are considered, the travel time between source and receiver can be determined [61]. The recorded traces provide a mean to measure the travel time of *P*-waves, to calculate the *P*-wave velocity, and to evaluate the corresponding modulus (if the density of the sample is known). Concerning the travel time and distance necessary to calculate the wave velocity, the determination of travel distance (distance between transducers) is generally considered as less problematic of the two. The determination of the travel time, on the other hand, is more controversial.

In figure 3, some characteristic features of the transmitted and received signal were marked. Feature A marks the impulse of the transmitted signal, and on the received signal the characteristic features are first-arrival B, first-local maximum C and first-local minimum D. Generally, the signal transmitted until the D-point is called the first event, which carries the essential information. The first arrival method calculates the time difference between the first peak in the transmitted signal (A) and the first deflection observed in the output signal (B). But the peak-peak method takes the time between the first peak (A) of the input signal and the first and/or second peak (C and/or D) of the received signal. Here, we pick consistent peak-peak travel time (difference between time of A and C), since selecting the first arrival pin is sometimes less straightforward [35].

The *P*-wave modulus *M* obtained by different travel time selection criteria is shown in figure 11 for samples at a hydrostatic stress state of *p* = 200 kPa. As was expected from figure 11, the results obtained using the first arrival point (B in figure 4) give lower values than the other two. It is noteworthy to mention that the qualitative trend of the *P*-wave modulus is similar for the three methods, as illustrated in figure 11.

## Appendix C

Complementary to the FFT analysis results, the energy is plotted for a sample with rubber content of *ν* = 0 at different confining pressures (from *p* = 50 to 500 kPa) in figure 12. This observation prevails despite the fact that the main frequency of glass media remains unchanged by increasing the confining pressure. Moreover, one can determine the main frequency of glass-dominated samples from this figure.
